# Tranexamic Acid in Combination With Vancomycin or Gentamicin Has a Synergistic Effect Against Staphylococci

**DOI:** 10.3389/fmicb.2022.935646

**Published:** 2022-06-30

**Authors:** Antonio Benjumea, Marta Díaz-Navarro, Rama Hafian, Emilia Cercenado, Mar Sánchez-Somolinos, Javier Vaquero, Francisco Chana, Patricia Muñoz, María Guembe

**Affiliations:** ^1^Department of Orthopaedic Surgery and Traumatology, Hospital General Universitario Gregorio Marañón, Madrid, Spain; ^2^Department of Clinical Microbiology and Infectious Diseases, Hospital General Universitario Gregorio Marañón, Madrid, Spain; ^3^Instituto de Investigación Sanitaria Gregorio Marañón, Madrid, Spain; ^4^School of Biology, Universidad Complutense de Madrid, Madrid, Spain; ^5^CIBER Enfermedades Respiratorias-CIBERES (CB06/06/0058), Madrid, Spain; ^6^School of Medicine, Universidad Complutense de Madrid, Madrid, Spain

**Keywords:** tranexamic acid, bacterial growth, antimicrobial effect, synergy, *in vitro* model

## Abstract

**Background:**

Tranexamic acid (TXA) is an antifibrinolytic agent applied in orthopedic surgery and has been proven to reduce post-surgery infection rates. We previously showed that TXA also had an additional direct antimicrobial effect against planktonic bacteria. Therefore, we aimed to evaluate whether it has a synergistic effect if in combination with antibiotics.

**Materials and Methods:**

Three ATCC and seven clinical strains of staphylococci were tested against serial dilutions of vancomycin and gentamicin alone and in combination with TXA at 10 and 50 mg/ml. The standardized microtiter plate method was used. Minimal inhibitory concentrations (MICs) were calculated by standard visualization of well turbidity (the lowest concentration at which complete absence of well bacterial growth was observed by the researcher) and using the automated method (the lowest concentration at which ≥80% reduction in well bacterial growth was measured using a spectrophotometer).

**Results:**

Tranexamic acid-10 mg/ml reduced the MIC of vancomycin and gentamicin with both the standard method (V: 1-fold dilution, G: 4-fold dilutions) and the automated turbidity method (vancomycin: 8-fold dilutions, gentamicin: 8-fold dilutions). TXA-50 mg/ml reduced the MIC of gentamicin with both the standard turbidity method (6-fold dilutions) and the automated turbidity method (1-fold dilutions). In contrast, for vancomycin, the MIC remained the same using the standard method, and only a 1-fold dilution was reduced using the automated method.

**Conclusion:**

Ours was a proof-of-concept study in which we suggest that TXA may have a synergistic effect when combined with both vancomycin and gentamicin, especially at 10 mg/ml, which is the concentration generally used in clinical practice.

## Introduction

Periprosthetic joint infections (PJIs) are caused by bacterial biofilm that adheres to the prosthesis and hampers eradication of infection once established (Josse et al., 2019; Shoji and Chen, 2020). Therefore, preventive strategies are necessary to reduce the frequency of PJIs at the time of surgery (Kapadia et al., 2016; Romanò et al., 2017).

Tranexamic acid (TXA) is an antifibrinolytic agent used in daily clinical practice as it binds to plasminogen and enables blood clotting (Jennings et al., 2016; Draxler et al., 2019). Numerous studies support the use of this drug in surgical interventions, specifically in orthopedic surgery and traumatology, as it has improved the postoperative management of patients undergoing both elective and emergency surgeries without leading to increase in complications among the healthy population (Melvin et al., 2015; Jennings et al., 2016; Waddell et al., 2016). Some authors hypothesize that as TXA reduces local hematoma, it could indirectly reduce infection rates (Ruder and Springer, 2017; Klement et al., 2020; Lacko et al., 2020; Sukeik et al., 2020; Yazdi et al., 2020).

The hypothesis of whether, in addition to its anti-fibrinolytic action, TXA could have a direct antibiotic effect similar to that observed with hyaluronic acid is an interesting area of research (Romanò et al., 2017). Therefore, given that this effect may be related to biofilm formation and have an antibacterial effect against planktonic cells under experimental conditions (Zhang et al., 2021; Benjumea et al., 2022), we assessed whether TXA could have a synergistic effect when in combination with vancomycin and gentamicin, which are two of the most commonly used antibiotics in orthopedic surgery (Ratto et al., 2016; Myers and Lipof, 2020; Indelli et al., 2021; Parvizi, 2021).

## Materials and Methods

Ours was an *in vitro* experimental study carried out at Hospital General Universitario Gregorio Marañón, Madrid, Spain (Supplementary Material 1; Behzadi and Gajdács, 2021).

We included 10 strains: three American Type Culture Collection (ATCC) strains (methicillin-susceptible *Staphylococcus aureus* [MSSA; ATCC29213], methicillin-resistant *Staphylococcus aureus* [MRSA; ATCC43300], and methicillin-resistant *Staphylococcus epidermidis* [MRSE; ATCC35984]), and seven high-biofilm-producing clinical strains of MSSA (*n* = 3), MRSA (*n* = 1), and MRSE (*n* = 3) isolated from patients with PJIs.

### Tranexamic Acid at Increasing Concentrations

We first tested serial dilutions of TXA from 5 to 50 mg/ml diluted in sterile water to identify which TXA concentration/s best reduced bacterial turbidity. The solutions were kept at 2–4°C until use.

The procedure was performed following CLSI guidelines and using a microtiter plate (Clinical and Laboratory Standards Institute, 2018). The wells of a 96-well plate were inoculated with 50 μl of a suspension of 10^6^ cfu/ml of each microorganism in Müller-Hinton broth and treated with serial dilutions (from 5 to 50 mg/ml) of TXA (50 μl of twice the final dose in the well). Positive controls (bacterial suspension) were treated with 100 μl of sterile water, and negative controls (only Müller-Hinton broth) were treated with 100 μl of TXA 50 mg/ml. All the experiments were performed in triplicate. Plates were incubated at 37°C for 24 h.

#### Calculation of Percentage Reduction in Turbidity

We used a spectrophotometer (492 nm) to calculate the percentage reduction in turbidity of each well at each TXA concentration compared to the positive control.

### Tranexamic Acid Combined With Vancomycin and Gentamicin

We then tested the effect of TXA at extreme concentrations where similar results were observed according to reduction in bacterial turbidity (10 and 50 mg/ml) in combination with serial dilutions of vancomycin (Merck KGaA, Darmstadt, Alemania) (from 0.03 to 16 mg/L) and gentamicin (Merck KGaA, Darmstadt, Alemania) (from 0.06 to 32 mg/L), which were also tested separately in parallel.

The procedure was performed following CLSI guidelines (32nd edition, 2018) and using a microtiter plate (Clinical and Laboratory Standards Institute, 2018). The wells of a 96-well plate were inoculated with 5 μl of a suspension of 10^7^ cfu/ml of each microorganism in a Müller-Hinton broth and treated with the following: 100 μl of vancomycin (from 0.03 to 16 mg/L) alone (100 μl) and in combination (50 μl of twice the final dose in the well) with 50 μl of TXA (at 10 and 50 mg/ml) and gentamicin (from 0.06 to 32 mg/l) alone (100 μl) and in combination (50 μl of twice the final dose in the well) with 50 μl of TXA (at 10 and 50 mg/ml). Positive controls (bacterial suspension) were treated with 100 μl of sterile water, and negative controls (only Müller-Hinton broth) were treated with 100 μl of each highest antibiotic concentration. All the experiments were performed in triplicate. Plates were incubated at 37°C for 24 h.

### Calculation of Minimal Inhibitory Concentration

We defined the Minimal Inhibitory Concentration (MIC) of each antibiotic tested, alone or in combination with TXA, using both the standard visualization of well turbidity and the automated turbidity method (Clinical and Laboratory Standards Institute, 2018).

For the standard visualization method, MIC was defined as the lowest concentration at which complete absence of well bacterial growth (compared to the positive control) was observed by the researcher (Clinical and Laboratory Standards Institute, 2018).

For the automated turbidity method, MIC was defined as the lowest concentration at which ≥80% reduction in well bacterial growth (compared to the positive control) was measured using a spectrophotometer at 492 nm.

As our objective was not to identify whether the strains were susceptible or resistant but to assess the possible synergistic effect of TXA, we did not take into consideration the lack of correlation between the MICs obtained by one method or the other. We used the automated turbidity method in addition to the standard visualization method, because it provided a higher MIC, which enabled us to better detect the synergistic effect of TXA starting from higher MICs than with the standard method.

### Data Analysis

The effect of serial dilutions of TXA against planktonic bacteria was expressed as the mean percentage reduction in well turbidity compared to the positive control.

Minimal Inhibitory Concentration was expressed as the geometric mean (MIC_50_) with the corresponding ranges.

Data were expressed by combining the results from all species together and separately according to species (*Staphylococcus aureus, N* = 6, and *Staphylococcus epidermidis, N* = 4).

The data are collected in the repository C.0001228 of the ISCIII.

## Results

### Percentage Reduction in Well Turbidity When Tranexamic Acid Serial Dilutions Were Tested Against Planktonic Bacteria

**FIGURE 1:**
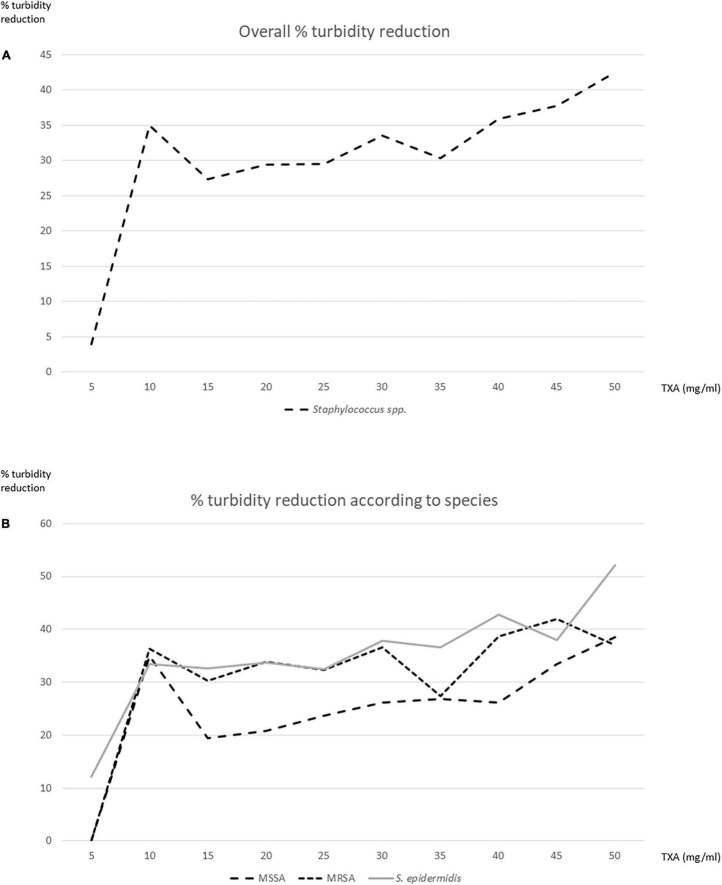
Percentage reduction in well turbidity after testing tranexamic acid (TXA) serial dilutions against planktonic bacteria. **(A)** Overall. **(B)** According to species.

Overall, TXA at 5 mg/ml showed almost no antibacterial effect (mean percentage reduction in turbidity 4%), especially for *S. aureus* (0%) (Figures 1A,B).

Above 5 mg/ml, the mean percentage reduction in well turbidity ranged from 27.4 to 42.5% (Figures 1A,B).

In order to select the extreme concentrations at which similar percentage reductions were observed, we tested TXA 10 (35%) and 50 mg/ml (42.5%) in subsequent experiments.

The mean percentage reduction in well turbidity for TXA 10 mg/ml in the *S. aureus* strains and the *S. epidermidis* strains was, respectively, 35.7 and 33.5%. The mean percentage reduction in well turbidity for TXA 50 mg/ml in the *S. aureus* strains and the *S. epidermidis* strains was, respectively, 37.8 and 52.2% (Figures 1A,B).

### Minimal Inhibitory Concentration of Vancomycin and Gentamicin Alone and in Combination With Tranexamic Acid Calculated Using the Standard Visualization Method

**FIGURE 2:**
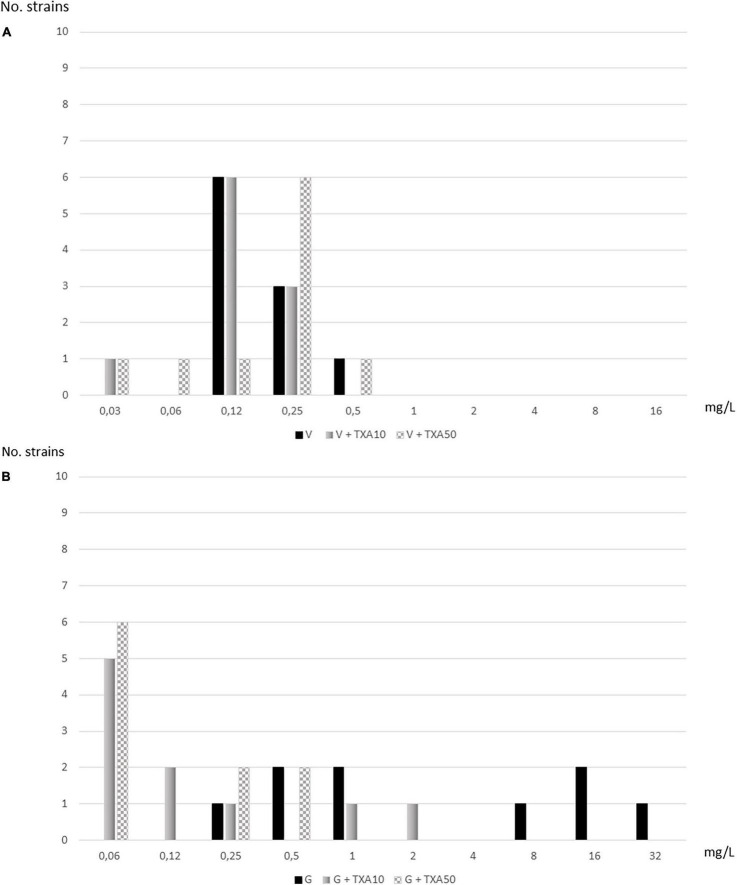
Distribution of strains according to serial dilutions of vancomycin and gentamicin alone and in combination with tranexamic acid (TXA) using the standard visualization method. **(A)** Vancomycin. **(B)** Gentamicin.

Using the standard visualization method, TXA-10 mg/ml reduced the MIC_50_ of vancomycin and gentamicin by 1- and 4-fold, respectively: TXA10-V, 0.12 mg/L vs. V, 0.25 mg/L and TXA10-G, 0.5 mg/L vs. G, 8 mg/L. TXA-50 mg/ml reduced the MIC_50_ of gentamicin (TXA50-G, 0.12 mg/L vs. G, 8 mg/L) by 6-fold, whereas the MIC_50_ of vancomycin remained unchanged (0.25 mg/L) (Table 1 and Figures 2A,B).

**TABLE 1:** MIC_50_ for vancomycin and gentamicin alone and in combination with tranexamic acid (TXA) using the standard visualization method.

Treatment	*Staphylococcus aureus* MIC_50_ (ranges) mg/L	MIC_50_ fold reductions (no.)	*Staphylococcus epidermidis* MIC_50_ (ranges) mg/L	MIC_50_ fold reductions (no.)	Overall MIC_50_ (ranges) mg/L	MIC_50_ fold reductions (no.)
Vancomycin	0.12 (0.12–0.25)		0.25 (0.12–0.5)		0.25 (0.12–0.5)	
Vancomycin + TXA 10 mg/ml	0.12 (0.12–0.25)	0	0.12 (0.03–0.25)	1	0.12 (0.03–0.25)	1
Vancomycin + TXA 50 mg/ml	0.25 (0.12–0.25)	NA*	0.25 (0.03–0.5)	0	0.25 (0.03–0.5)	0
Gentamicin	4 (0.25–16)		16 (0.5–32)		8 (0.25–32)	
Gentamicin + TXA 10 mg/ml	0.12 (0.06–0.25)	5	1 (0.06–2)	4	0.5 (0.06–2)	4
Gentamicin + TXA 50 mg/ml	0.12 (0.06–0.5)	5	0.25 (0.06–0.5)	6	0.12 (0.06–0.5)	6

TXA, tranexamic acid; MIC, minimal inhibitory concentration; NA, not applicable. *1-fold increase was observed.

### Minimal Inhibitory Concentration of Vancomycin and Gentamicin Alone and in Combination With Tranexamic Acid Calculated Using the Automated Turbidity Method

**FIGURE 3:**
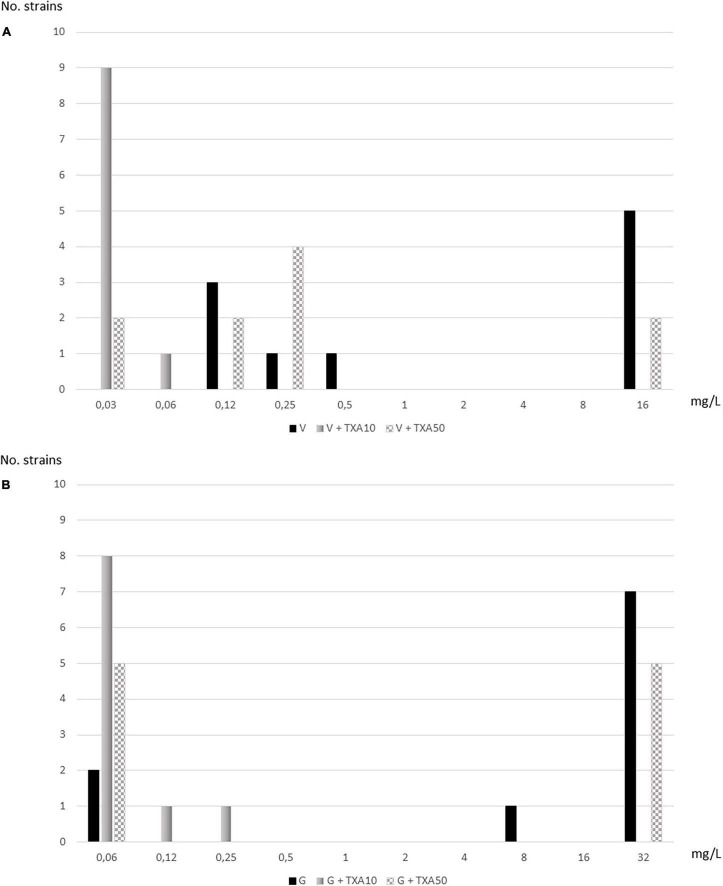
Distribution of strains according to serial dilutions of vancomycin and gentamicin alone and in combination with tranexamic acid (TXA) using the automated turbidity method. **(A)** Vancomycin. **(B)** Gentamicin.

Using the automated turbidity method, TXA-10 mg/ml reduced the MIC_50_ of vancomycin and gentamicin by 8-fold: TXA10-V, 0.03 mg/L vs. V, 8 mg/L and TXA10-G, 0.12 mg/L vs. G, >32 mg/L. TXA-50 mg/ml reduced the MIC50 of vancomycin and gentamicin 1-fold: TXA50-V, 4 mg/L vs. V, 8 mg/L and TXA50-G, 16 mg/L vs. G, >32 mg/L (Table 2 and Figures 3A,B).

**TABLE 2:** MIC_50_ for vancomycin and gentamicin alone and in combination with tranexamic acid (TXA) using the automatized turbidity method.

Treatment	*Staphylococcus aureus* MIC_50_ (ranges) mg/L	MIC_50_ fold reductions (no.)	*Staphylococcus epidermidis* MIC_50_ (ranges) mg/L	MIC_50_ fold reductions (no.)	Overall MIC_50_ (ranges) mg/L	MIC_50_ fold reductions (no.)
Vancomycin	4 (0.12–16)		16 (16–16)		8 (0.12–16)	
Vancomycin + TXA 10 mg/ml	0.03 (0.03–0.06)	8	0.03 (0.03–0.03)	8	0.03 (0.03–0.06)	8
Vancomycin + TXA 50 mg/ml	0.12 (0.03–0.25)	1	8 (0.03–16)	1	4 (0.03–16)	1
Gentamicin	16 (0.06–32)		32 (32–32)		32 (0.06–32)	
Gentamicin + TXA 10 mg/ml	0.06 (0.06–0.12)	7	0.12 (0.06–0.25)	8	0.12 (0.06–0.25)	8
Gentamicin + TXA 50 mg/ml	8 (0.06–32)	1	16 (0.06–32)	1	16 (0.06–32)	1

*TXA, tranexamic acid; MIC, minimal inhibitory concentration.*

## Discussion

In combination with vancomycin and gentamicin, TXA showed a significant synergistic effect against the staphylococcal strains.

As we previously demonstrated that the effect of TXA on reduction in PJI rates was due to its direct effect by reducing bacterial growth (Benjumea et al., 2022), in addition to its indirect effect by reducing hematoma (Lohinai et al., 2015; Ruder and Springer, 2017; Draxler et al., 2019; Perez-Roman et al., 2019; Klement et al., 2020; Lacko et al., 2020; Sukeik et al., 2020; Yazdi et al., 2020), we further investigated whether it could also have a synergistic effect if in combination with antibiotics. Therefore, we decided to test it with vancomycin and gentamicin, which are two of the most commonly used antibiotics in orthopedic surgery (Ratto et al., 2016; Gandhi et al., 2018; Myers and Lipof, 2020; Indelli et al., 2021; Parvizi, 2021).

We also previously observed that percentage reductions in bacterial growth were generally better when the concentration of TXA was lower (Benjumea et al., 2022). Therefore, in the present study, we tested the effect of TXA at serial dilutions. We demonstrated that the percentage reduction in well turbidity was similar between TXA concentrations of 10 and 50 mg/ml (range 35–42.5%). Also interesting was the finding that TXA at 5 mg/ml did not have an antimicrobial effect (4% reduction in well turbidity). Thus, we confirm that if the final volume of TXA was finally reached and absorbed in the joint did not reach at least 10 mg/ml, TXA would not have any antimicrobial effect at all. However, this is not a likely scenario, as the dose of TXA used by most authors in the literature is between 1 and 3 g. Therefore, if the joint volume of one knee is estimated at 131 (±53) ml, the intra-articular TXA dilution would be approximately 10–30 mg/ml (Hewitt and Stringer, 2008; Maniar et al., 2012; Matziolis et al., 2015; Jennings et al., 2016).

Although toxicity has been reported at TXA concentrations of >20 mg/ml (Parker et al., 2018; Sukur and Kucukdurmaz, 2018; McLean et al., 2019; Mortazavi et al., 2020; Bolam et al., 2021; González Osuna et al., 2021; Ma et al., 2021; Touzopoulos et al., 2021), we decided to include a concentration of 50 mg/ml in our experiments in order to demonstrate that the synergistic effect was not dose-dependent. Using the standard visualization method, TXA 50 mg/ml reached higher fold reductions in gentamicin MIC_50_ in *S. epidermidis* (6 vs. 4 with TXA 10 mg/ml), although the same fold reduction in gentamicin MIC_50_ was observed for *S. aureus* (5 with TXA 10 and 50 mg/ml). Moreover, no synergistic effect was observed when TXA 50 mg/ml was combined with vancomycin on *S. aureus* or on *S. epidermidis*, whereas TXA 10 mg/ml reduced by 1-fold the vancomycin MIC_50_ in *S. epidermidis*. In addition, using the automated turbidity method, TXA 50 mg/ml reduced both the vancomycin and gentamicin MIC_50_ only by 1-fold compared with the 8-fold reduction for vancomycin and gentamicin with TXA 10 mg/ml.

Regarding the possible risk of using an anti-fibrinolytic drug in combination with an antibiotic during staphylococcal infection, to our knowledge, there are no studies answering the question of whether TXA combined with an antibiotic could produce or increase an adverse effect other than that which these drugs can produce by themselves. However, in orthopedic surgery, the use of systemic antibiotic treatment as prophylaxis of periprosthetic infection is widespread, including the use of antibiotic-loaded cement. On the other hand, TXA is commonly used both systemically and locally concomitantly in the same surgeries. In our clinical experience, we have not found an increase in local and/or systemic adverse effects (Wong et al., 2010; Poeran et al., 2014; Franchini et al., 2018).

Therefore, we can conclude that TXA 10 mg/ml showed a better synergistic effect with either vancomycin or gentamicin than TXA 50 mg/ml. However, whether the TXA mechanisms of action are direct or indirect is still unknown. Some studies have tried to answer this question. On the one hand, there is research that indicates the inhibitory effect of TXA, a lysine analog, on certain enzymes that favors biofilm formation (Lohinai et al., 2015). On the other hand, Draxler et al. (2019) conclude in their study on TXA and immune response that TXA has an immunomodulatory effect, altering the study of plasma cytokine levels, the number of immune cells, and the expression of multiple immunological markers.

The main limitation of the study is that we only tested 6 and 4 strains of *S. aureus* and *S. epidermidis*, respectively. Future studies are needed to validate our findings in a large sample size of clinical staphylococci strains. Furthermore, as we only tested a single dose, we do not know whether continuous doses could affect our results, as observed in the *in vivo* study by Zhang et al. (2021). In addition, we did not run growth curves or time-kill kinetic assays.

## Conclusion

Our results suggest that TXA may reduce the MIC of vancomycin and gentamicin against staphylococci in an experimental planktonic model at the concentration generally used in clinical practice (10 mg/ml). This proof-of-concept study provides new insights regarding the preventive role of TXA in clinical practice.

## Data Availability Statement

The raw data supporting the conclusions of this article will be made available by the authors, without undue reservation.

## Author Contributions

MG and FC were responsible for the organization and coordination of the trial. FC, JV, PM, and MG were the chief investigator and responsible for the data analysis. AB, MD-N, RH, EC, and MS-S developed the trial design and data collection. All authors contributed to wrote the final manuscript and approved the submitted version.

## Conflict of Interest

The authors declare that the research was conducted in the absence of any commercial or financial relationships that could be construed as a potential conflict of interest.

## Publisher’s Note

All claims expressed in this article are solely those of the authors and do not necessarily represent those of their affiliated organizations, or those of the publisher, the editors and the reviewers. Any product that may be evaluated in this article, or claim that may be made by its manufacturer, is not guaranteed or endorsed by the publisher.
