# Corrigendum: Tranexamic acid in combination with vancomycin or gentamicin has a synergistic effect against staphylococci

**DOI:** 10.3389/fmicb.2024.1355087

**Published:** 2024-02-02

**Authors:** Antonio Benjumea, Marta Díaz-Navarro, Rama Hafian, Emilia Cercenado, Mar Sánchez-Somolinos, Javier Vaquero, Francisco Chana, Patricia Muñoz, María Guembe

**Affiliations:** ^1^Department of Orthopaedic Surgery and Traumatology, Hospital General Universitario Gregorio Marañón, Madrid, Spain; ^2^Department of Clinical Microbiology and Infectious Diseases, Hospital General Universitario Gregorio Marañón, Madrid, Spain; ^3^Instituto de Investigación Sanitaria Gregorio Marañón, Madrid, Spain; ^4^School of Biology, Universidad Complutense de Madrid, Madrid, Spain; ^5^CIBER Enfermedades Respiratorias-CIBERES (CB06/06/0058), Madrid, Spain; ^6^School of Medicine, Universidad Complutense de Madrid, Madrid, Spain

**Keywords:** tranexamic acid, bacterial growth, antimicrobial effect, synergy, *in vitro* model

In the published article, there were errors in Table 2 as published. The *S. aureus* MIC_50_ fold reductions for gentamicin+TXA 50 mg/ml appeared as 5. This has been corrected to 1. The *S. epidermidis* MIC_50_ fold reductions for gentamicin+TXA 10mg/ml appeared as 9. This has been corrected to 8. The corrected Table 2 and its caption appear below.

**Table 2:** MIC_50_ for vancomycin and gentamicin alone and in combination with TXA using the automatized turbidity method.

**Treatment**	***S. aureus* MIC_50_ (ranges) mg/L**	**MIC_50_ fold reductions (no.)**	***S. epidermidis* MIC_50_ (ranges) mg/L**	**MIC_50_ fold reductions (no.)**	**Overall MIC_50_ (ranges) mg/L**	**MIC_50_ fold reductions (no.)**
Vancomycin	4 (0.12–16)		16 (16–16)		8 (0.12–16)	
Vancomycin+TXA 10 mg/ml	0.03 (0.03–0.06)	8	0.03 (0.03–0.03)	8	0.03 (0.03–0.06)	8
Vancomycin+TXA 50 mg/ml	0.12 (0.03–0.25)	1	8 (0.03–16)	1	4 (0.03–16)	1
Gentamicin	16 (0.06–32)		32 (32–32)		32 (0.06–32)	
Gentamicin+TXA 10 mg/ml	0.06 (0.06–0.12)	7	0.12 (0.06–0.25)	8	0.12 (0.06–0.25)	8
Gentamicin+TXA 50 mg/ml	8 (0.06–32)	1	16 (0.06–32)	1	16 (0.06–32)	1

TXA, tranexamic acid; MIC, minimal inhibitory concentration.

The authors apologize for these errors and state that they do not change the scientific conclusions of the article in any way. The original article has been updated.

