# Supplementary figures and images for: Tranexamic Acid in Combination With Vancomycin or Gentamicin Has a Synergistic Effect Against Staphylococci
Source: Front Microbiol. 2022 Jun 30;13:935646. doi: 10.3389/fmicb.2022.935646 (PMC9280180; doi:10.3389/fmicb.2022.935646)

**Supplemental data file. Flowchart of the experimental design**


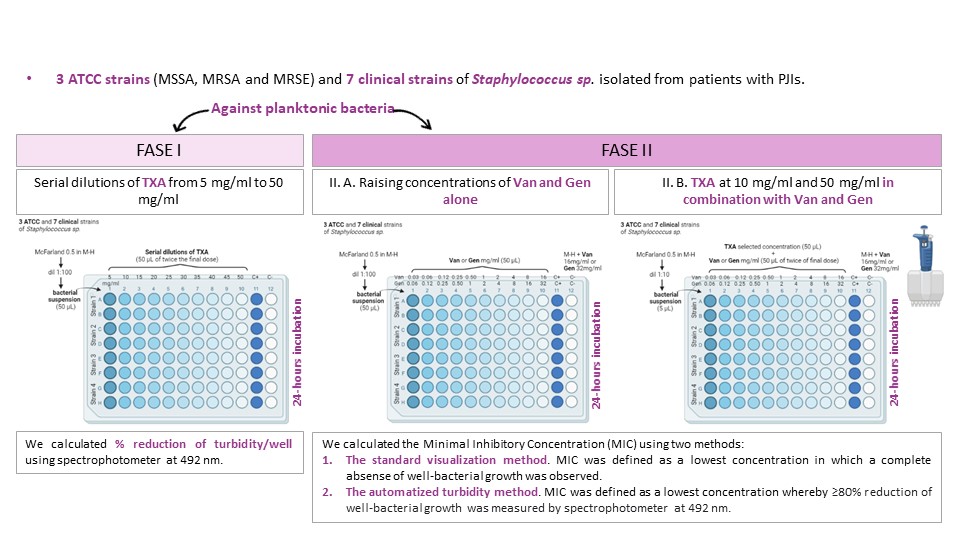

Supplement: Supplementary file 1 [file Data_Sheet_1.docx]
